# Direct and indirect risk associated with the use of dietary supplements among persons with dementia in a Norwegian memory clinic 

**DOI:** 10.1186/s12906-017-1765-5

**Published:** 2017-05-12

**Authors:** Hilde Risvoll, Trude Giverhaug, Kjell H. Halvorsen, Marit Waaseth, Frauke Musial

**Affiliations:** 1 NKS Kløveråsen AS, Junkernveien 67, 8076, Bodø, Norway; 2 RELIS North Norway, University Hospital North Norway, Sykehusvegen 38, 9019 Tromsø, Norway; 3 Department of Pharmacy, UiT The Arctic University of Norway, pb 6050 Langnes, 9037 Tromsø, Norway; 4 NAFKAM, UiT The Arctic University of Norway, pb 6050 Langnes, 9037 Tromsø, Norway

**Keywords:** Patient safety, Dementia, Dietary supplements, Risk management, Direct risk, Indirect risk, Drug interactions, Caregivers, Home care services, Cross-sectional survey

## Abstract

**Background:**

The use of dietary supplements (DS) is common among persons with dementia. Direct risks associated with DS use include adverse events and DS-drug interactions. A direct risk is a risk caused by the treatment itself. Indirect risks are related to the treatment setting, such as the conditions of use, and not to the treatment itself. Because dementia symptoms may reduce a person’s ability to cope with the administration of DS, the use of DS may pose a threat to safety as an indirect risk. The aim of this study was to describe the extent of DS use among persons with dementia in ambulatory care and to identify some relevant direct and indirect risks related to DS use.

**Methods:**

We conducted a survey among 151 persons with dementia attending an outpatient memory clinic in Northern Norway. Study measurements included: the participants’ characteristics, cognitive functioning, functioning in the activities of daily living (ADL), and the use of DS and prescription drugs (PD). We assessed direct risks by evaluating potential DS-drug interactions and indirect risks by evaluating the conditions under which it was used.

**Results:**

Forty-six percent (*n* = 70) of the persons with dementia used DS. Ninety-seven percent (*n* = 147) used PD. We found potentially clinically relevant DS-drug interactions representing a direct risk in eight persons with dementia (11% of users). While only 36% (*n* = 26) of the participants received assistance with the administration of DS, 73% (*n* = 106) received assistance with the administration of PD. Persons with dementia living alone were at risk of not receiving assistance, as home care service seldom was involved in DS administration. Data indicated that assistance with DS administration was not provided for all persons with dementia in need, representing an indirect risk to these persons. Only one-third of the persons with dementia and half of the caregivers were aware of the general risks of adverse events and interactions associated with the use of DS.

**Conclusions:**

Persons with dementia use DS frequently, yet DS use may be associated with direct and indirect risks to patient safety as potentially clinically relevant interactions were discovered and DS intake often was unsupervised.

## Background

Dementia is a general term for progressive diseases that lead to loss of mental abilities interfering with and causing problems in the activities of daily living (ADL). Alzheimer’s disease is the most common form of dementia, and memory problems are the most common symptom [1]. Persons affected by dementia become increasingly dependent on assistance throughout the course of the disease. Because a considerable number of single persons with dementia continue to live by themselves for quite some time, they become increasingly dependent on home care services. Today, only symptomatic treatment is available for Alzheimer’s disease [1], resulting in a search for alternative treatments by persons with dementia and their caregivers. Several dietary supplements (DS) on the market claim to improve memory problems, but the scientific evidence is sparse [2–5]. Prevalence estimates of DS use in persons with dementia range from 27% to 58% [6–10]. The variation in estimates could be due to heterogeneity in study design, including the number of participants, the time period of interest and the types of DS studied.

“Is this Dietary Supplement that my spouse is using, safe? Can he take it together with his prescription drugs?” Medical doctors often receive these types of questions and they rarely have straightforward answers. Living with persons with dementia can be quite challenging, and caregivers often find themselves unable to control the situation [11]. For example, one daughter found half-empty pillboxes containing dietary supplements (DS), prescription drugs (PD) and over the counter (OTC) drugs all around her mother’s apartment, even inside the microwave oven. Similar examples are well known among caregivers of persons with dementia and these types of questions and worries prompted the conduct of this study. DS are often labeled as “natural” and are therefore regarded as safe products by many consumers. DS can nonetheless cause harm through adverse events [12, 13], and even lethal cases have occurred [14, 15]. Their potential to interact with PD is also of concern [16, 17].

A direct risk is a risk related to the treatment itself such as adverse events and DS-drug interactions [18, 19]. Moreover, there are other threats to patient safety from DS use, such as variability in quality and content; for example, adulterants in the form of pharmaceuticals have been found [20, 21]. Another concern is the profound lack of studies documenting safety, tolerability, and efficacy [22]. Another safety issue is a striking lack of reliable information about DS-drug interactions [23, 24]. This, together with the risk of overdosing or forgetting to take the daily dose of treatment, poses a considerable threat to patient safety. Factors which are not directly related to the DS itself, are often referred to as “indirect risk factors.” By definition, indirect risks are risks related to the treatment setting, instead of the treatment itself [18, 19]. Indirect risks are often caused by acts of omission and can include obtaining insufficient information about the patients’ medical history, inadequacies in diagnostic testing, as well as persons not receiving needed drugs or not receiving adequate help with the administration of their drugs [25]. Indirect risks from the use of complementary and alternative medicine (CAM) in general can also include delayed diagnosis and a lack of awareness among CAM practitioners of the therapeutic limitations of CAM [19, 26].

**Fig. 1:**
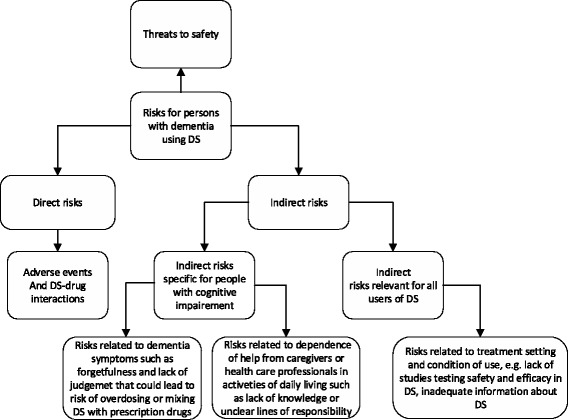
Risks related to use of dietary supplements in persons with dementia. *Abbreviation*: *DS*, dietary supplements

With regard to persons with dementia, disease-related cognitive problems may increase the indirect risks from DS usage (Fig. 1).

Forgetfulness and impaired judgment may lead to erroneous use of DS (or PD and OTC); for instance, persons with dementia may forget that they have already taken their daily dose of tablets, or they use several DS with the same ingredients. Moreover, persons with dementia may confuse DS with PD, leading to errors in the administration of both. Loss of initiative can prevent persons with dementia from discussing DS use with their family physician or from obtaining reliable information about DS at the pharmacy or on the internet. Their reduced capability to identify and express their own signs and symptoms can prevent persons with dementia, especially in the advanced stages of the disease, from disclosing actual adverse events of DS and PD. Moreover, studies report that few persons with dementia disclose their use of DS to health care personnel [6–8]. This indirect risk situation is particularly threatening because several indirect risk factors may lead to overdosing; which invariably increases direct risks such as increased toxicity.

The degree to which persons with dementia are exposed to indirect risks related to the use of DS is currently unknown. The lack of awareness and knowledge about risks from DS among caregivers and health care professionals, represent an indirect risk to the persons with dementia [27]. This is also the case of indistinct lines of responsibility. In particular, little is known about the involvement of home care services in administering and dispensing DS to persons with dementia who still live in their homes. However, home care services visit persons with dementia in their homes, and can therefore, potentially, be the part of the healthcare system that has the best possibilities to safeguard those who have decided to use DS. Indirect risks related to health care personnel’s professional conduct may be an accessible window for intervention within the risk structure of DS use in persons with dementia.

The aim of this study was to describe the extent of DS use among persons with dementia in ambulatory care and to identify direct and indirect risks related to DS use. More specifically, we wanted to investigate whether persons with dementia received assistance with the administration of DS and PD and relate this to vulnerability factors in these persons.

## Methods

### Study population

We conducted a questionnaire-based survey of persons with dementia attending an outpatient memory clinic in North Norway from November 2011 to end of October 2013. We included all consecutive patients who met the ICD-10 criteria for dementia and who visited a neurologist (HR) for a regular neurological follow-up. To ensure reliable responses, only persons with dementia who were accompanied by a caregiver, who could supply information, were included. If the person with dementia brought several caregivers, the one closest to the person with dementia was defined as the main caregiver. For two persons who brought no relatives, health care professionals familiar with the persons were defined as caregivers. We excluded four persons with dementia because of severe communication problems with their caregivers. The caregivers were not assessed, but the communication problems were judged to be caused by cognitive impairment, most likely in combination with profound hearing loss, which was not properly compensated by a hearing aid. The numbers of patient visits to the clinic before inclusion varied, as did the type of dementia.

### Survey development and implementation

The study was initiated after several caregivers had raised concerns about DS-drug interactions and about incorrect use of DS by their relatives with dementia. Therefore, these topics were our main concern. HR and TG constructed the questionnaire based on previous studies and on their experience from clinical practice at a memory clinic and from a drug information center, respectively. We strived for simple wording and open-ended questions. The feasibility of the instrument was tested on five persons with dementia prior to the start of the study. Some parts of the current survey were part of the routine consultation such as age, gender, whether the patients lived alone, whether they received help from homecare, MMSE-NR and RDRS-2, list of PD and OTC and whether the person with dementia received help with the administration of PD. Twelve additional questions were designed exclusively for this survey. The patient/caregiver received altogether 16 questions, nine open-ended and seven yes/no questions. Seven of these questions are not included in this publication because the content of data would exceed the scope of one article. These data will be published later. All participants were asked about their current use of DS, and users were further asked to specify the product names and where they had procured their DS (pharmacy, merchandiser/retailer of dietary supplements, grocery store, internet or telephone sale, direct from CAM therapist). We also asked who had initiated the use (patient themselves, spouse, other relatives, health care personnel, retailers), and who secured correct administration (patient themselves, spouse or relatives, home care service). We asked all persons with dementia and all caregivers if they knew that dietary supplements might have potential negative effects such as adverse events and interactions with prescription drugs. This was a general question and not related specifically to the DS used by some of the participants.

It was important for us to involve the persons with dementia themselves as much as possible. We therefore designed the questionnaire as a structured face-to-face interview where both the person with dementia and his or her caregiver were present during the interview. The interviews were performed by HR. If the participants did not understand the questions as they were read out, additional explanations were given. The definition of DS (e.g., that it includes herbs, vitamins, minerals and other compounds, or a mixture of different ingredients) was explained to all participants. They were also given examples of common brand names of DS during the interview. A non-judgmental, open attitude toward the use of DS was maintained during the interview. In most cases, both the persons with dementia and the caregivers provided the answers, but in cases of severe dementia, it was mostly the caregivers who answered the questions. If persons with dementia and caregivers provided divergent answers or if they both reported uncertainty, we asked them to check at home and contact us later by telephone. When there was persistent disagreement/uncertainty, the answers were left blank. The response was oral and the answers were written down and categorized.

### Cognitive assessment

All persons with dementia were assessed using the Mini-Mental Status Examination-Norwegian Revision (MMSE-NR) [28, 29], and, with the assistance of the caregivers, using the Rapid Disability Rating Scale-2 (RDRS-2) [30]. The aim was to collect up-to-date information about persons with dementias’ cognitive- and ADL functions. The RDRS-2 scale ranges from 21 to 84 points; a score of 21 indicate normal ADL function, while a score of 84 indicates complete dysfunctionality. MMSE-NR screens people for difficulties in cognitive function with scores ranging from 0 to 30. A score below 24 is suggestive of cognitive problems, such as in dementia, but can also be caused by other reasons; values in the range of 25–27 might represent early stages of dementia. Scores above 28 indicate normal function with some exceptions such as frontotemporal dementia in the initial stages of the disease [31]. Persons with higher education could achieve higher MMSE scores even in the presence of dementia [32]. We did not assess for educational level.

### Direct risk assessment

Direct risk of DS is harm caused by the products themselves. In this study, we assessed only DS-drug interactions and not adverse events. Lists of individual persons’ PD, DS and OTC were collected and sent anonymously to the Regional Pharmacovigilance Center North Norway (RELIS North Norway) for assessment of DS-drug interactions. The Natural Medicines Comprehensive Database, Medline and the Norwegian RELIS database were used to identify potential clinically relevant DS-drug interactions. Due to time constraints during the interview, we made no assessment of potential clinical correlations from potential DS-drug interactions. The survey took place in an outpatient clinic setting and it was too time consuming to assess DS–drug interactions during the consultation. As several of the participants had travelled quite a distance to get to the clinic, we did not include another patient visit in the survey. Our outpatient clinic covers a wide geographical area including several ferry routes, with the longest traveling distance of nearly 400 km. The memory clinic contacted the home care service or the family physician in most cases to obtain an updated list of the persons with dementia’s PD and OTC.

### Indirect risk assessment

Indirect risks from DS use are related to the condition of use. In this study, we investigated to what degree persons with dementia received assistance administering DS, in general and according to their cognitive function. We also investigated the knowledge of risks, and whether knowledge of risks influenced the use of DS, or help with administering DS.

### Definition of dietary supplements

We used the definition of DS from the Dietary Supplement Health and Education Act of 1994 paragraph 3a [33]. The definition states that a DS is a product containing one or more of the following: “a vitamin, a mineral, an herb or other botanical, an amino acid, or a dietary substance for use by man to supplement the diet by increasing the total dietary intake; or a concentrate, metabolite, constituent, extract, or combination of these ingredients.” We excluded pure vitamin supplements that were used to treat a diagnosed deficiency. We also excluded untreated edible oils, herbs used as spices, food bars and beverages such as teas. This was because our main interest was in supplements that could be confused with drugs by the persons with dementia and administered by home care services. None of the participants reported using supplements in other administration forms than tablets.

### Ethics

The study was approved by the Regional Committee for Medical and Health Research Ethics North, reference number: 2011/1705. An employee at the outpatient clinic, who was not involved in patient care, presented the study details and obtained written consent from each participant and caregiver before consultation and data collection.

### Statistics

Data were analyzed using IBM SPSS (Statistical Package for the Social Sciences) version 22.0 (IBM Corporation, Armonk, NY, US) for Windows. We present descriptive statistics such as absolute and relative frequencies, means and standard deviations. We applied an independent Student’s t-test for continuous variables and Pearson’s chi-square or Fisher’s exact tests for categorical variables. We used logistic regression for binary data to analyze the associations between the frequency of persons with dementia receiving assistance with DS administration and the initiators of DS use in these persons. Significance level was set at 5% and was adjusted for multiple testing according to Bonferroni.

## Results

### Use of DS

We included 151 persons with dementia, mean age was 73.3 years (range 20–90), average MMSE-NR was 19.6 (range 0–29). The youngest participant had genetically and clinically verified Juvenile Huntington’s disease. The person with MMSE-NR score of 29 had the diagnosis frontotemporal dementia. Sixty-three percent were women and 32% lived alone. All responders were of Scandinavian heritage. The caregivers were mostly spouses (51%) or children (35%), and more seldom other relatives, friends or health care professionals (14%). Three persons declined to join the study. Twelve other persons were excluded for different reasons (e.g. HR judged the persons with dementia as being too exhausted). The overall response rate was 90%.

Seventy persons with dementia (46%) reported the use of DS. On average, they used 1.7 DS (range 1–6). Fish oils were the most commonly used DS (40 persons, 57%), followed by various mixed herbal supplements (29 persons, 41%) and vitamin and mineral supplements (28 persons, 40%). Thirty-two (46%) of the users consumed more than one DS product.

As Table 1 shows, the users and non-users of DS were similar with regard to age, gender, living conditions, and use of PD in general and dementia drugs in particular. Even though users of DS showed less severe reduction in cognitive function measured by MMSR-NR and a trend towards better ADL functioning measured by RDRS-2 compared with non-DS-users, both groups showed clear signs of cognitive impairment.

**Table 1:** Comparison between users and non-users of dietary supplements

Persons with dementia’s characteristics	Users of DS		Non-Users of DS			Total population	
	*n* = 70		*n* = 81		*p*-value	*n* = 151	
Age, year (mean (±SD))	72.7	(11.2)	73.7	(9.8)	0.547	73.3	(10.4)
Women (n (%))	49.0	(70.0)	46.0	(56.8)	0.094	95.0	(62.9)
Living alone (n (%))	23.0	(32.9)	25.0	(30.9)	0.793	48.0	(31.8)
Home care services (n (%))	31.0	(44.3)	33.0	(40.7)	0.660	64.0	(42.4)
Numbers of PD (mean (±SD))	4.7	(3.4)	4.4	(2.6)	0.582	4.6	(3.0)
Persons using dementia drugs (n (%))	29.0	(41.4)	41.0	(50.6)	0.259	70.0	(46.4)
Numbers of OTC *(mean (±SD))	0.8	(0.8)	0.7	(0.7)	0.334	0.7	(0.7)
MMSE-NR score (mean (±SD))	21.7	(4.5)	17.8	(6.3)	**<0.001**	19.6	(5.8)
RDRS-2 score (mean (±SD))	34.5	(8.8)	38.5	(11.5)	0.019	36.7	(10.5)

*Abbreviations*: *DS* dietary supplements, *SD* standard deviation, *PD* prescription drug, *OTC* over-the-counter drug, *MMSE-NR* Mini Mental State Examination-Norwegian Revision, *RDRS-2* Rapid Disability Rating Scale-2

*Data are missing from two persons

The RDRS-2 scale range from 21 to 84, where a score of 21 points indicates normal function in activities of daily living and a score of 84 points indicate complete dysfunctionality. The MSEE-NR scale range from zero to 30, where 30 points indicate normal cognitive function. Statistics are independent Student’s t-test for continuous variables such as age, numbers of PD and OTC, MMSE-NR and RDRS-2. Statistics are Pearson’s chi-square or Fisher’s exact tests for categorical variables such as gender, living alone, receiving help from home care service and using dementia drugs. Bonferroni adjusted α was 0.05 / 9 resulting in α < 0.006. Significant comparisons after adjustment are printed bold

In most cases, the persons with dementia did not initiate the use of DS. In 20 cases (29%) the persons with dementia took the initiative themselves, while in 15 cases (22%) the spouse took the initiative, in 14 cases (20%) other relatives, in 10 cases (14%) health care personnel, and in 10 cases (14%) DS-retailers took the initiative. Data were missing from one participant.

Persons with dementia had purchased DS on the internet or through telephone sale in 26 cases (37%), at pharmacies in 25 cases (36%), at DS-retailers in 16 cases (23%), and at grocery stores in 13 cases (19%). Some persons had purchased their DS at several places.

In three cases (4%), a relative provided the DS for free. Two of these relatives were DS retailers.

### Direct risks related to use of DS

Of the 147 persons with dementia who used PD, two of these only used vitamin B12 injections every third month. On average, the persons used 4.6 PD (range 0–17) and 0.7 OTCs (range 0–3). We identified potentially clinically relevant interactions between DS and PD/OTCs in eight persons (11%). In four persons these interactions involved anticoagulants, and in four persons antihypertensives. *Boswellia serrata, Vaccinium macrocarpon* and omega-3 could possibly interact with warfarin. Atenolol was combined with lutein, *Camellia sinensis*, *Bacopa monnieri, Capsicum annum, Crocus sativus* and procyanidolic oligomers. Amlodipine was combined with astaxanthin, *Panax ginseng, Punica granatum,* lutein and *Boswellia serrate*. Metoprolol was combined with pomegranate, *Cordyceps sinensis* and *Panax ginseng.* One participant suffered from tachycardia, which could have been negatively affected by her DS use. She used a DS (in tablet formulation) containing *Camellia sinensis* among several other ingredients*. Camellia sinensis* contains high amounts of caffeine and also theophylline [34], which may lead to tachycardia. At the same time, this person used a beta-blocker for her tachycardia. In this case we recommended that the use of that particular DS was ended. In addition, one participant used DS causing a daily intake of vitamin D, chromium and copper above the recommended dietary intake, RDI [35, 36].

### Indirect risks related to use of DS

Only 26 out of 70 persons with dementia (37%) received assistance administering their DS, compared to 106 persons out of 145 (73%) who received assistance administering their PD. Two persons in the PD group did not depend on daily assistance, as their only medications were vitamin B12 injections every third month. Living alone was associated with not receiving assistance with DS; this was not the case for persons with dementia who used PD (Table 2). Persons with dementia who received assistance with PD had lower MMSE-NR scores and higher RDRS-2 scores as an indication of more advanced dementia. After the Bonferroni correction, this difference was no longer statistically significant for MMSE-NR score in participants who received assistance with DS, although there was a significant difference in their RDRS-2 score.

**Table 2:** Characteristics of adults with dementia receiving assistance with dietary supplements or prescription drugs

	Assistance with DS		No assistance with DS			Assistance with PD		No assistance with PD		
Numbers of persons with dementia	*n* = 26		*n* = 44		*p*-value	*n* = 106**		*n* = 39**		*p*-value
Age, year (mean (±SD))	76.8	(8.1)	70.3	(12.1)	0.017	74.5	(10.9)	70.4	(8.9)	0.040
Women (n (%))	16.0	(61.5)	33.0	(75.0)	0.235	65.0	(61.3)	27.0	(69.2)	0.380
Living alone (n (%))	3.0	(11.5)	20.0	(45.5)	**0.004**	35.0	(33.0)	10.0	(25.6)	0.394
Home care services (n (%))	14.0	(53.8)	17.0	(38.6)	0.216	64.0	(60.4)	0.0	-	**<0.001**
Numbers of PD (mean (±SD))	5.5	(2.9)	4.3	(3.7)	0.161	5.4	(2.9)	3.0	(2.4)	**<0.001**
Persons using dementia drugs (n (%))	12.0	(46.2)	17.0	(38.6)	0.537	54.0	(50.9)	16.0	(41.0)	0.289
Numbers of OTC (mean (±SD))*	0.7	(0.8)	0.8	(0.8)	0.604	0.7	0.7	1.0	0.7	0.047
MMSE-NR (mean (±SD))	20.1	(4.2)	22.6	(4.5)	0.021	18.5	(6.1)	22.4	(4.2)	**<0.001**
RDRS-2 (mean (±SD))	38.9	(9.5)	32.0	(7.3)	**0.001**	40.2	(10.0)	27.7	(5.3)	**0.001**

*Abbreviations: DS* dietary supplements, *PD* prescription drug, *SD* standard deviation, *OTC* over-the-counter drug, *MMSE-NR* Mini Mental State Examination-Norwegian Revision, *RDRS-2* Rapid Disability Rating Scale-2

*Data are missing from two participants

**Six respondents did not use PD regularly. Four respondents used no PD. Two respondents, who used only vitamin B12 injection, were not included in the 145 respondents that used PD, as they were independent on daily assistance

Note that the RDRS-2 scale range from 21 to 84, where a score of 21 points indicates normal function in activities of daily living and a score of 84 points indicate complete dysfunctionality. The MSEE-NR scale range from zero to 30, where 30 points indicate normal cognitive function

Statistics are independent Student’s t-test for continuous variables such as age, numbers of PD and OTC, MMSE-NR and RDRS-2. Statistics are Pearson’s chi-square or Fisher’s exact tests for categorical variables such as gender, living alone, receiving help from home care service and using dementia drugs. Bonferroni adjusted α was 0.05/9 resulting in α ≤ 0.006. Significant comparisons after adjustment are printed bold

Several persons who did not receive assistance with DS and PD had MMSE-NR and RDRS-2 scores indicating that it was questionable whether they were able to handle the administration of DS and PD on their own. The lowest MMSE-NR score was 13 in both groups, and the highest RDRS-2 score was 45 in participants who did not receive assistance with the administration of DS, and 39 in participants who did not receive assistance with PD. Fifty percent of the 44 persons with dementia who administered their DS themselves had MMSE scores below 24 points. Fifty-seven percent of the 39 persons with dementia who administered their PD themselves had MMSE scores below 24 points.

Two out of twelve persons with dementia who used anticoagulants did not receive assistance with drug administration. Both were in an early stage of dementia as measured by MMSE-NR and RDRS-2 (lowest MMSE-NR score was 20, highest RDRS-2 score was 30). One of these persons also used digoxin, three antihypertensives and a DS containing *Boswellia serrata*, astaxanthin and omega-3-fatty acid.

**Fig. 2:**
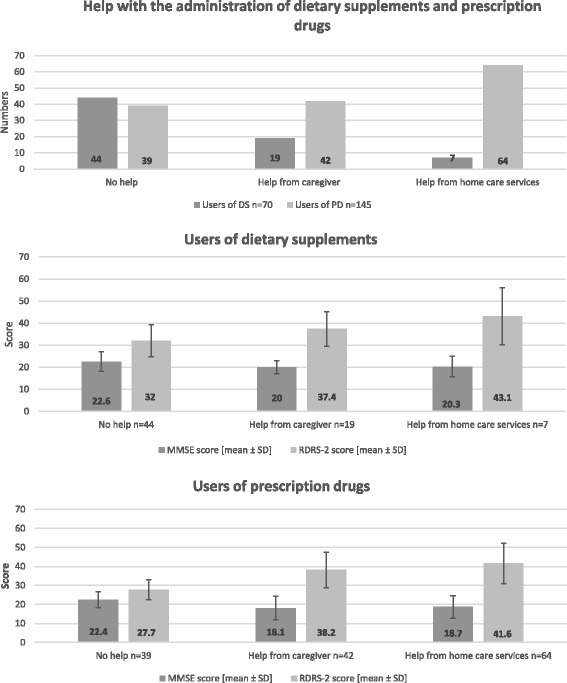
Assistance with administration of dietary supplements and prescription drugs related to function. *Abbrevation*: *DS*, dietary supplements; *PD*, prescription drugs; *ADL*, activities of daily living; *MMSE-NR*, Mini Mental State Examination-Norwegian Revision; *RDRS-2*, Rapid Disability Rating Scale-2; *SD*, standard deviation. Two persons with dementia, who used only vitamin B12 injection, were not included in the 145 persons that used PD, as they were not dependent of daily assistance. Note that Fig. 2 demonstrate descriptive data. The RDRS-2 scale range from 21 to 84, where a score of 21 points indicates normal function in activities of daily living and a score of 84 points indicate complete dysfunctionality. The MSEE-NR scale range from zero to 30, where 30 points indicate normal cognitive function

Caregivers were most frequently assisting with DS, and home care services with PD. Home care services were seldom involved in assisting persons with dementia with the administration of DS (Fig. 2). In 17 cases, persons with dementia received home care services for PD, without the home care service being involved in the administration of these persons’ DS (Table 2). A direct comparison between those who received help with the administration of DS and those who received help with the administration of PD can not be made because of overlap between those two populations.

Spouses and other relatives were the most frequent initiators of DS use. There was a relationship between who initiated use and receiving assistance with DS. When spouses or health care personnel initiated DS use, persons with dementia were more likely to receive assistance with the administration of the DS (Table 3).

**Table 3:** The relationship between assistance with DS administration and who initiated the use of DS calculated by logistic regression analysis

Initiator of DS use	Receiving assistance with DS					
	Yes	No	Total	*p*-value	OR	95% CI
Persons with dementia themselves	4	16	20	0.50	Ref	Ref
Spouses	10	5	15	**<0.01**	6.40	1.47–27.83
Other relatives	2	12	14	0.50	0.53	0.09–3.24
Health care personnel	8	2	10	**<0.01**	12.80	2.02–81.12
Retailers	1	9	10	0.38	0.04	0.04–3.54
Total	25	44	69*			

*Abbreviations: DS* dietary supplements, *OR* odds ratio, *CI* confidence interval

*Data are missing from one participant. Statistics are logistic regression for binary outcome

Significant﻿﻿ results are printed in bold. We used an alpha level of 0.05 to evaluate statistical significance

### Awareness of risk

Forty-eight persons with dementia (32%) and 77 caregivers (51%) said they were aware that use of DS might increase a general risk for adverse events and interactions with PD. Data were missing from four participants. Participants’ DS use did not differ depending on knowledge of risk among caregivers and persons with dementia. In the 78 cases where caregivers knew about the risk for adverse events or DS-drug interactions, 35 persons with dementia used DS (44%); in the 69 cases where caregivers were not aware, 33 (48%) used DS (*p* = 0.720, Pearson’s χ^2^ 0.129, df 1). Among persons with dementia who used DS, caregivers’ knowledge of risk did not influence help with administration of DS. If caregivers knew about the risk for adverse events or DS-drug interactions, 16 out of 35 (46%) users of DS received help with the administration; if caregivers were not aware, 10 out of 33 (30%) users of DS received help with the administration, (*p* = 0.19, Pearson’s χ^2^ 1.708, df 1).

## Discussion

The use of DS was common among persons with dementia. With regard to direct risks, only a minority of the persons with dementia were aware of the potential risk of adverse events and/or interactions from DS. Although only few persons with dementia used combinations suggesting clinically relevant DS-drug interactions, the ones we found were potentially harmful.

The persons with dementia did not receive the same degree of assistance with their DS as with their PD. Two thirds of the persons received assistance with the administration of their PD, while only one third received assistance with the administration of DS. Additionally, home care services were minimally involved in DS monitoring.

### Use of DS

No previous studies have addressed the use of DS in persons with dementia in a Norwegian setting, but the estimated prevalence of DS use among Norwegians in general ranges from 44% to 74% [37, 38]. Almost half of the persons with dementia in the current study reported the use of DS, which is consistent with studies on persons with dementia from Canada, Germany, India and the US [6–10] and also consistent with the use of DS in the general Norwegian population [38].

The only difference between users of DS and non-users were signs of better cognitive function in users. The higher frequency of DS use among persons with less advanced dementia, indicated by higher MMSE-NR and a trend towards lower RDRS-2 scores, is in line with previous research [9]. Our cross-sectional analysis does not allow for causal interpretations. Possible explanations for the result could be that some DS actually slow down cognitive decline (although not scientifically documented), or that people with dementia tend to stop using DS as the disease progresses or a combination of both. Reasons for discontinuing a DS could be that persons with dementia stop buying it because of increased forgetfulness or loss of initiative. Other reasons might be increasing reluctance to take DS or tablets in general, or that the persons with dementia and their caregivers lose faith in DS, if the effect that they hoped for fails.

### Direct risks of DS use

The direct risks caused by potentially clinically relevant DS-drug interactions in 11% in the DS users gives reason for concern. The concurrent use of several similar DS products, such as several fish oils by some of the persons with dementia, in combination with anticoagulants, should be mentioned. Although a risk of increased bleeding from taking omega-3-fatty-acid supplements has been suggested, excessive bleeding due to inhibition of platelet function has not been demonstrated in clinical studies [39]. The clinical importance of combining omega-3-fatty acids and drugs that increase the risk of bleeding (e.g., anticoagulants and aspirin) is debated. Several of the participants used more than one product containing vitamin D, and the total intake exceeded the RDI of this vitamin. The clinical relevance of this, however, is uncertain because the given RDI of vitamin D is an estimate which is well below toxic amounts [36]. Nevertheless, extra caution should be taken in persons with dementia, as they may be more susceptible to overdosing because they may not take their DS as intended.

Other studies have reported potentially clinically relevant DS-drug interactions in 10–40% of DS users [10, 38, 40]. As the types of DS and the types of drugs used by different populations could vary over time, a direct comparison between studies is difficult. As DS are regulated differently than PD, several factors lead to lack of knowledge about potential interaction between DS and drugs. Pharmacovigilance challenges regarding DS include lack of studies documenting safety and tolerability, and underreporting of suspected adverse events [22].

### Indirect risks of DS use

Not surprisingly, persons with dementia who had higher RDRS-2, and lower MMSE-NR scores received more assistance with the administration of PD. Assistance with DS was related to higher RDRS-2 score, but not to lower scores in the MMSE-NR. Persons with dementia received less assistance when it came to the administration of DS compared to PD. The persons with dementia who used DS had slightly better cognitive functioning than non-users, and we cannot exclude that this could have affected how much assistance these persons received. The differences in the cognitive test scores were rather small, but statistically and clinically relevant [41]. However, better cognitive functioning cannot be the sole explanation, as some of these persons received assistance with PD but not with DS. Several studies have set an MMSE score below 24 as a threshold for persons who could have trouble with self-administration of drugs [42, 43]. Although both MMSE-NR scores and RDRS-2 scores are rough estimates, the scores of the persons with dementia who did not receive assistance with the administration of DS or PD indicate that several of these persons were in need of assistance. The fact that probably most of the persons with dementia were in need of assistance with both DS and PD/OTC, and a relatively large proportion of the participants did not receive this assistance, is, in our opinion, the key message from this study.

Persons with dementia living alone are at a particular risk of not receiving assistance with DS, as home care services seldom assisted these persons with DS even though they frequently assisted with PD. We found that when health care personnel were the initiators, more persons with dementia received assistance administering DS. This suggests that health care personnel’s lack of awareness of persons with dementias’ DS use is a key factor in why assistance is not given.

Studies have reported that users of DS rarely inform health care personnel about their use [6–8, 38]. A likely reason for this is the belief that the supplements are harmless [44]. Most of the persons with dementia and half of the caregivers in the current study were unaware that DS might cause adverse events and DS-drug interactions. Caregivers’ knowledge of the risks of adverse events and DS-drug interactions did not influence patient’s use of DS or assistance with the administration of DS. We have not investigated the reasons for actions or omissions on the part of persons with dementia or their caregivers. It is possible that caregivers believe the benefits from DS outweigh the disadvantages, and that the potential risk of DS use is too small to necessitate precautions. Optimistic bias, the belief that one is less likely than one’s peer to suffer harm, can also lead to the denial of risks [45].

Inadequate adherence to the administration of DS, PD and OTC challenge patient safety and requires risk management [46]. The degree of adherence generally declines with increasing number of tablets to be taken [46]. We found that DS users consumed on average 2.1 tablets more per day than non-users (7.2 vs. 5.1 tablets, respectively). Adherence might therefore be a safety issue of special concern among DS users.

Although this study focused on DS, it is important to keep in mind that PD might cause far more damage than DS when taken incorrectly [47]. It is of concern that two participants who used anticoagulants, one of them also digoxin, lacked assistance with the administration of their PD.

In this study, more than one third of the persons with dementia bought their DS at pharmacies. Pharmacy employees possess knowledge of DS and can inform and advise persons with dementia and their caregivers. Nonetheless, the majority purchased their DS outside of the traditional health care service and could therefore not expect any guidance.

### Strengths and limitations of the study

The participation rate in the study was high (90%). All participants were included prospectively and consecutively from an unselected dementia population, with a minimum set of exclusion criteria, to reduce selection bias and maintain external validity. The dementia population in this study was comparable to dementia populations in other studies with regard to age, gender distribution and level of cognitive function [48]. Our study population is different from populations in studies done in more ethnically diverse countries, by being ethnically homogeneous. This is not due to selection, but to a high degree of ethnic homogeneity in the elderly age groups in our geographic region [49]. Moreover, our findings are not necessarily generalizable to persons with dementia who were never referred to specialized health care, and persons with dementia who do not have a caregiver, as these groups were not included in our study. The participants were recruited from a Norwegian outpatient clinic and the results should be interpreted on the background of the particularities of the Norwegian healthcare system. However, the general problem of direct and indirect risks associated with DS use in persons with dementia will probably be relevant in other health care structures as well.

The study measurements included clinical assessment as well as face-to-face interviews. When it comes to clinical assessment, we assessed ADL function by the RDRS-2 scale, because this scale was part of the routine assessment in the memory clinic. The fact that this scale rarely is used in research is a limitation of our study. Thus, RDRS-2 scale gives a description of ADL function without giving us the opportunity to compare our results to other studies. As some of the drug lists were unreconciled, our approach of contacting home care services and family physicians ensured data quality. Some underreporting of DS use may have occurred, and our reported prevalence of use is therefore a conservative estimate. Furthermore, our study population was small, even though comparable to earlier prevalence studies.

### Practical implications

Persons with dementia are particularly vulnerable, as the dementia symptoms reduce their ability to take care of themselves. It is therefore important to take any increased direct or indirect risk seriously. Health care personnel and family physicians in particular, should be aware that around half of the persons with dementia use DS. Particular emphasis should be placed on persons with dementia who live alone and persons with dementia in earlier disease stages, as these subgroups could be less likely to receive assistance with the administration of DS. Another concern is co-use of DS, anticoagulants, and other drugs with a narrow therapeutic window, in which DS-drug interactions may have serious clinical consequences.

Caregivers of persons with dementia living alone might buy and initiate the use of DS without being able to assist in their administration or to be able to ensure safe use. It may therefore be advisable for family physicians and home care services to discuss DS with caregivers, particularly when the persons with dementia live alone.

In order to ensure patient safety, we suggest formalizing the assistance provided by the health care services related to DS. Conduction of risk assessment including evaluation of DS-drug interaction should, in our opinion, be mandatory. Both pharmacists and family physicians are qualified to take on the assignment. Distinct lines of responsibility, pointed out by health authorities, would probably increase patient safety. If the use of DS is safe and to be continued, health care personnel should secure assistance with DS administration for persons with dementia in need of assistance. As we observed, there is a lack of knowledge of the potential risks concerning DS use among persons with dementia and their caregivers, thus we suggest that more information is made available to the public about DS.

## Conclusions

The use of dietary supplements was common in the dementia population studied and several sources of direct and indirect risks were identified. The sources of the increased risk give reason for concern, and might also be relevant to other groups of vulnerable persons with mental or functional challenges, such as old age frailty, intellectual disability or severe mental illness.

## Abbreviations

ADL: Activities of daily living
CAM: Complementary and alternative medicine
CI: Confidence interval
DS: Dietary supplements
MMSE-NR: Mini Mental State Examination-Norwegian Revision
OR: Odds ratio
OTC: Over-the-counter drugs
PD: Prescription drugs
RDI: Recommended dietary intakes
RDRS-2: Rapid Disability Rating Scale −2
RELIS: Regional pharmacovigilance center
SD: Standard deviation

## References

[1] Winblad B, Amouyel P, Andrieu S, Ballard C, Brayne C, Brodaty H (2016). Defeating Alzheimer's disease and other dementias: a priority for European science and society. Lancet Neurol.

[2] Sydenham E, Dangour AD, Lim WS (2012). Omega 3 fatty acid for the prevention of cognitive decline and dementia. Cochrane Database Syst Rev.

[3] Persson T, Popescu BO, Cedazo-Minguez A (2014). Oxidative stress in Alzheimer's disease: why did antioxidant therapy fail?. Oxidative Med Cell Longev.

[4] Obermann KR, Morris JC, Roe CM (2013). Exploration of 100 commonly used drugs and supplements on cognition in older adults. Alzheimers Dement.

[5] Birks J, Grimley Evans J (2009). *Ginkgo biloba* for cognitive impairment and dementia. Cochrane Database Syst Rev.

[6] Dhikav V, Anand KS (2012). Complementary and alternative medicine usage among Alzheimer's disease patients. Int Psychogeriatr.

[7] Hogan DB, Ebly EM (1996). Complementary medicine use in a dementia clinic population. Alzheimer Dis Assoc Disord.

[8] Landin J, Frolich L, Schwarz S (2008). Use of alternative therapies in patients with dementia and mild cognitive impairment: a prospective, controlled study. International Journal of Geriatric Psychiatry.

[9] Coleman LM, Fowler LL, Williams ME (1995). Use of unproven therapies by people with Alzheimer's disease. J Am Geriatr Soc.

[10] Dergal JM, Gold JL, Laxer DA, Lee MS, Binns MA, Lanctot KL (2002). Potential interactions between herbal medicines and conventional drug therapies used by older adults attending a memory clinic. Drugs Aging.

[11] Laporte Uribe F, Heinrich S, Wolf-Ostermann K, Schmidt S, Thyrian JR, Schafer-Walkmann S, et al. Caregiver burden assessed in dementia care networks in Germany: findings from the DemNet-D study baseline. Aging Ment Health. 2016:1–12. doi:10.1080/13607863.2016.1181713. 10.1080/13607863.2016.1181713 27171484

[12] Bunchorntavakul C, Reddy KR (2013). Review article: herbal and dietary supplement hepatotoxicity. Aliment Pharmacol Ther.

[13] Ernst E (1998). Harmless herbs? A review of the recent literature. Am J Med.

[14] Kupiec T, Raj V (2005). Fatal seizures due to potential herb-drug interactions with *Ginkgo biloba*. J Anal Toxicol.

[15] Chan TY (2012). Contributory factors in herb-induced fatal aconite poisoning. Forensic Sci Int.

[16] Shalansky S, Lynd L, Richardson K, Ingaszewski A, Kerr C (2007). Risk of warfarin-related bleeding events and supratherapeutic international normalized ratios associated with complementary and alternative medicine: a longitudinal analysis. Pharmacotherapy.

[17] Hu Z, Yang X, Ho PC, Chan SY, Heng PW, Chan E (2005). Herb-drug interactions: a literature review. Drugs.

[18] Wardle JL, Adams J (2014). Indirect and non-health risks associated with complementary and alternative medicine use: An integrative review. European Journal of Integrative Medicine.

[19] Stub T, Salamonsen A, Kristoffersen A, Musial F (2015). How to handle worsening of condition during treatment - risk assessment in homeopathic practice. Forsch Komplementmed.

[20] Chan K (2003). Some aspects of toxic contaminants in herbal medicines. Chemosphere.

[21] Vaclavik L, Krynitsky AJ, Rader JI (2014). Mass spectrometric analysis of pharmaceutical adulterants in products labeled as botanical dietary supplements or herbal remedies: a review. Anal Bioanal Chem.

[22] Shaw D, Ladds G, Duez P, Wiliamson E, Chan K (2012). Pharmacovigilance of herbal medicine. J Ethnopharmacol.

[23] Owens C, Baergen R, Puckett D (2014). Online sources of herbal product information. Am J Med.

[24] Raynor DK, Dickinson R, Knapp P, Long AF, Nicolson DJ (2011). Buyer beware? Does the information provided with herbal products available over the counter enable safe use?. BMC Med.

[25] Hayward RA, Asch SM, Hogan MM, Hofer TP, Kerr EA (2005). Sins of omission: getting too little medical care may be the greatest threat to patient safety. J Gen Intern Med.

[26] Ernst E (2001). Complementary medicine: its hidden risks. Diabetes Care.

[27] Kemper KJ, Gardiner P, Gobble J, Woods C (2006). Expertise about herbs and dietary supplements among diverse health professionals. BMC Complement Altern Med.

[28] Folstein MF, Folstein SE, McHugh PR (1975). "Mini-mental state". A practical method for grading the cognitive state of patients for the clinician. J Psychiatr Res.

[29] Strobel C, Engedal K: MMSE-NR2 (MMS - norsk revisjon) (article in Norwegian language). Available at: file:///C:/Users/uin/Downloads/Manual%20MMSE-NR.pdf Assessed: 10 May 2016.

[30] Linn MW, Linn BS (1982). The rapid disability rating scale-2. J Am Geriatr Soc.

[31] Krueger CE, Bird AC, Growdon ME, Jang JY, Miller BL, Kramer JH (2009). Conflict monitoring in early frontotemporal dementia. Neurology.

[32] Crum RM, Anthony JC, Bassett SS, Folstein MF (1993). Population-based norms for the Mini-Mental State Examination by age and educational level. JAMA.

[33] U.S. Department of Health & Human Services (1994). Dietary Supplement Health and Education Act of 1994. Public Law No. 103–417. Paragraph 3a. Health NIo, vol. 2016.

[34] Khurshid Z, Zafar MS, Zohaib S, Najeeb S, Naseem M (2016). Green Tea (*Camellia sinensis*): Chemistry and Oral Health. Open Dent J.

[35] Inrstitute of Medicine Panel on M (2001). Dietary Reference Intakes for Vitamin A, Vitamin K, Arsenic, Boron, Chromium, Copper, Iodine, Iron, Manganese, Molybdenum, Nickel, Silicon, Vanadium, and Zinc.

[36] Vieth R (2007). Vitamin D toxicity, policy, and science. J Bone Miner Res.

[37] Waaseth M, Nakling M, Bakken K, Grimsgaard S (2010). Use of dietary supplements and medication among postmenopausal women with vasomotor symptoms. Climacteric.

[38] Djuv A, Nilsen OG, Steinsbekk A (2013). The co-use of conventional drugs and herbs among patients in Norwegian general practice: a cross-sectional study. BMC Complement Altern Med.

[39] Wachira JK, Larson MK, Harris WS: n-3 Fatty acids affect haemostasis but do not increase the risk of bleeding: clinical observations and mechanistic insights. Br J Nutr. 2014; 111(9):1652-1662. doi:10.1017/s000711451300425x. 10.1017/S000711451300425X 24472372

[40] Bush TM, Rayburn KS, Holloway SW, Sanchez-Yamamoto DS, Allen BL, Lam T et al: Adverse interactions between herbal and dietary substances and prescription medications: a clinical survey. Altern Ther Health Med. 2007;13(2):30-35. Retrieved from http://search.proquest.com/docview/204836174?accountid=26469. 17405676

[41] Stein J, Luppa M, Maier W, Wagner M, Wolfsgruber S, Scherer M, Kohler M, Eisele M, Weyerer S, Werle J et al: Assessing cognitive changes in the elderly: reliable change indices for the Mini-Mental State Examination. Acta Psychiatr Scand. 2012;126(3):208-218. 10.1111/j.1600-0447.2012.01850.x 22375927

[42] Sinclair AJ, Girling AJ, Bayer AJ: Cognitive dysfunction in older subjects with diabetes mellitus: impact on diabetes self-management and use of care services. All Wales Research into Elderly (AWARE) Study. Diabetes Res Clin Pract. 2000;50(3):203-12. doi:10.1016/S0168-8227(00)00195-9. 10.1016/s0168-8227(00)00195-9 11106835

[43] Gellad WF, Grenard JL, Marcum ZA: A systematic review of barriers to medication adherence in the elderly: looking beyond cost and regimen complexity. Am J Geriatr Pharmacother. 2011; 9(1):11-23. doi:10.1016/j.amjopharm.2011.02.004. 10.1016/j.amjopharm.2011.02.004 PMC3084587 21459305

[44] Awad A, Al-Shaye D: Public awareness, patterns of use and attitudes toward natural health products in Kuwait: a cross-sectional survey. BMC Complement Altern Med. 2014;14:105. doi:10.1186/1472-6882-14-105. 10.1186/1472-6882-14-105 PMC3999934 24646341

[45] Weinstein ND, Klein WM. Resistance of personal risk perceptions to debiasing interventions. Health Psychol. 1995;14(2):132-40. doi:10.1037/0278-6133.14.2.132. 10.1037//0278-6133.14.2.132 7789348

[46] Maher RL, Hanlon J, Hajjar ER: Clinical consequences of polypharmacy in elderly. Expert Opin Drug Saf. 2014;13(1):57-65. doi:10.1517/14740338.2013.827660. 10.1517/14740338.2013.827660 PMC3864987 24073682

[47] Stocks SJ, Kontopantelis E, Akbarov A, Rodgers S, Avery AJ, Ashcroft DM. Examining variations in prescribing safety in UK general practice: cross sectional study using the Clinical Practice Research Datalink. BMJ. 2015;351. doi:10.1136/bmj.h5501. 10.1136/bmj.h5501 PMC4632209 26537416

[48] Meeuwsen EJ, Melis RJ, Van Der Aa GC, Goluke-Willemse GA, De Leest BJ, Van Raak FH et al. Effectiveness of dementia follow-up care by memory clinics or general practitioners: randomised controlled trial. BMJ. 2012;344:e3086. doi:10.1136/bmj.e3086. 10.1136/bmj.e3086 PMC3352696 22589500

[49] Henriksen K, Østby L, Ellingsen D: Immigration and immigrants 2010. Available at http://www.ssb.no/a/english/publikasjoner/pdf/sa122/sa122_en.pdf. Accessed 1 May 2017.

